# Clinical predictors of right upper paraesophageal lymph node metastasis from papillary thyroid carcinoma

**DOI:** 10.1186/1477-7819-10-164

**Published:** 2012-08-16

**Authors:** Yong-Seok Kim, Woo-Chan Park

**Affiliations:** 1 Department of Surgery, College of Medicine, The Catholic University of Korea, Yeouido St.Mary’s Hospital, 62 Yeouido-dong, Youngdeungpo-gu, Seoul 150-713, Korea

**Keywords:** Paraesophageal lymph node, Thyroid cancer, Papillary thyroid carcinoma

## Abstract

**Background:**

Central and lateral lymph node metastases are quite common in patients with papillary thyroid carcinoma, and the predictors for those metastases have been well studied. Right upper paraesophageal lymph node metastasis has rarely been studied. The aim of this study was to identify the clinicopathological characteristics that may be risk factors for right upper paraesophageal lymph node metastasis in patients with papillary thyroid carcinoma.

**Methods:**

This was a prospective observational study of 243 patients with papillary thyroid carcinoma (PTC) who underwent total thyroidectomy and comprehensive central lymph node dissection with or without lateral lymph node dissection between April 2008 and January 2010. The clinicopathologic findings from these patients were investigated and the patterns of lymph node metastasis were analyzed in the patients who had right upper paraesophageal lymph node disease.

**Results:**

Of the 243 patients undergoing lymph node dissection, 14 had right upper paraesophageal lymph node metastases. Two of these patients had right upper paraesophageal lymph node metastasis only, without central compartment metastasis. Univariate analysis of clinicopathologic findings showed that right upper paraesophageal lymph node metastasis had significant association with larger primary tumors, multifocal tumors, extrathyroid extension, and lymphatic invasion (*p <*0.05 for each factor).

**Conclusions:**

Although there were no independent predictors of right upper paraesophageal lymph node metastasis, it can be the only site of metastasis without other compartmental metastasis. Therefore, during surgery for patients with central or lateral lymph node metastases from PTC, it may be helpful to examine the right upper paraesophageal lymph nodes.

## Background

Papillary thyroid carcinoma (PTC) is the most common form of thyroid cancer. It generally has a favorable prognosis. As PTC tends to recur in a predictable locoregional pattern, appropriate surgical intervention [1] can be expected to increase long-term survival.

Cervical lymph node metastases are quite common in PTC and have been found in 20% to 50% of patients. Lymph node metastases are known to be important prognostic factors for locoregional and distant metastasis [2-4]. Local recurrence in the central compartment after initial surgery has been reported to increase mortality rates [5].

Total thyroidectomy is the standard procedure for all PTC >10 mm in size [3,6], but optimum treatment for smaller tumors and indications for lymph node dissection remain controversial. Lymph node metastases generally spread sequentially from the nodes of the central compartment of the neck to the nodes of the lateral compartment, and, in frequently, skip metastasis occurs [7-10]. The central compartment of the neck includes the precricoid (Delphian), pretracheal, and paratracheal lymph nodes. The right upper paraesophageal lymph nodes are difficult to distinguish from the central compartment; however, that section is important, because metastasis to a lateral lymph node can involve the mediastinum and because they are located close to the right recurrent laryngeal nerve [11,12]. The management of right upper paraesophageal lymph node metastases, particularly posterior to the right recurrent laryngeal nerve, remains unclear. Therefore, this study was performed to examine the clinicopathologic factors associated with right upper paraesophageal lymph node metastasis in patients with papillary thyroid carcinoma.

## Methods

A total of 243 consecutive patients with PTC were prospectively enrolled in this study and underwent thyroid surgery plus cervical lymph node dissection at Yeouido St. Mary’s Hospital of the Catholic University in Seoul, Korea, between April 2008 and January 2010. All patients underwent preoperative thyroid function tests, thyroid ultrasonography (US), US-guided fine-needle aspiration cytology (FNAC) of the thyroid nodules, and neck computed tomography (CT) for preoperative staging of PTC and evaluation of lymph node status.

Total thyroidectomy and central lymph node dissection were performed with or without lateral lymph node dissection. Surgery was performed when the thyroid nodules detected by US were proven to be papillary carcinoma on FNAC or when the FNAC was suspicious for malignancy. A frozen section biopsy of the thyroid lesion was performed during surgery. If frozen section results confirmed malignancy, total thyroidectomy and comprehensive central lymph node dissection were performed. Lateral lymph node dissection was performed if preoperative imaging studies were suspicious for metastasis, for instance, if the ultrasound image revealed loss of central hilum echogenicity, microcystic changes, or microcalcifications. Neck CT with and without intravenous contrast agent was performed preoperatively, and 5-mm image slices were obtained from the base of the skull to the thoracic inlet. A single radiologist evaluated the images for metastases in the lateral lymph nodes (LNs) of the neck. In patients whose CT scans showed strong enhancement, calcifications, or necrotic or cystic changes after intravenous contrast, lateral lymph node dissection was performed with correlation to the US images.

**Figure 1:**
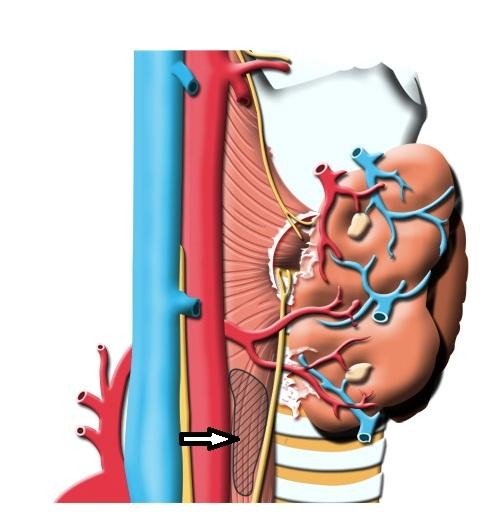
The arrow indicates the area that was dissected (right upper paraesophageal lymph node dissection).

The central compartment of the neck includes the region between the common carotid arteries and is bounded superiorly by the hyoid bone. The inferior border has been variably defined as the sternal notch or the innominate artery. The paratracheal nodes may be anterior and posterior to the recurrent laryngeal nerves. These nodes may be found posterior to the common carotid artery on the right because of the anatomic variation of the sides. In this study, dissection of the right upper paraesophageal lymph nodes included the area posterior to the right recurrent laryngeal nerve. The field was inspected for macroscopic disease before dissection. Figure1 shows the boundaries of the upper paraesophageal area that was dissected.

The histopathology of the thyroid tumor was determined from all biopsy specimens examined by the hospital’s pathologists. Pathologic reports were compiled, and clinicopathological characteristics, including tumor size, tumor location, multifocal tumor, tumor encapsulation, extrathyroidal extension, capsular invasion, lymphatic invasion, perineural invasion, vascular invasion, and concurrent thyroiditis were recorded.

Statistical analysis was performed using SPSS, version 18.0 (SPSS Inc., Chicago, IL). Univariate analysis was performed using the chi-square test and Fisher’s exact test, and multivariate analysis was performed using logistic regression analysis. Differences were considered significant for *p <*0.05.

This study was reviewed and approved by the institutional review board at Yeouido St. Mary’s Hospital.

## Results

The clinicopathologic characteristics of the 243 patients [200 females (82.3%) and 43 males (17.7%), mean age 49.54 years] are summarized in Table1. All patients underwent total thyroidectomy. The mean tumor size was 1.06 ± 0.60 cm. Tumors were located in the right lobe, left lobe, isthmus, and bilateral lobes in 41.6%, 32.1%, 2.4%, and 23.9% of patients, respectively. There were 37 patients with the primary lesion located in the upper third of the thyroid lobe, 81 patients with lesions in the middle third, and 43 patients with lesions in the lower third. Multifocal tumors were identified in 34.2% of patients. Positive nodal metastases occurred in 49.4% of patients. Among the 243 patients who underwent central neck dissection, 14(5.8%) patients were positive for right upper paraesophageal lymph node metastases.

**Table 1:** Clinicopathologic characteristics of 243 patients who underwent total thyroidectomy and cervical lymph node dissection

**Variables**		**Results(*n*** **= 243)**
Age	<45	88(36.2%)
	≥45	155(63.8%)
Gender	Male	43(17.7%)
	Female	200(82.3%)
Tumor size	<1 cm	128(52.7%)
	≥1 cm	115(47.3%)
Tumor location	Left	78(32.1%)
	Right	101(41.6%)
	Isthmus	6(2.4%)
	Both	58(23.9%)
Solitary lesion	Upper	37(15.2%)
	Middle	81(33.3%)
	Lower	43(17.7%)
	Isthmus	6(2.5%)
Multifocal lesion	Both lobes	58(23.9%)
	Affected lobe	14(5.8%)
Multiplicity	(−)	160(65.8%)
	(+)	83(34.2%)
Extrathyroidal extension	(−)	101(41.6%)
	(+)	142(58.4%)
Node metastasis	(−)	123(50.6%)
	(+)	120(49.4%)
Right upper paraesophageal metastasis	(−)	229(94.2%)
	(+)	14(5.8%)
Retrieved central lymph nodes	Mean(95%CI^a^)	8.3210(7.6611-8.9808)
Metastatic central lymph nodes	Mean(95%CI^a^)	1.5556(1.2071-1.9040)
Retrieved lateral lymph nodes	Mean(95%CI^a^)	19.8333(17.2426-22.4241)
Metastatic lateral lymph nodes	Mean(95%CI^a^)	1.9167(1.3089-2.5244)

^a^CI: confidence interval.

In 1 of the 14 patients with right upper paraesophageal lymph node metastasis, the other compartments were negative for lymph node metastasis. There was one patient with right upper paraesophageal lymph node metastasis and lateral lymph node metastasis. Eight of the 14 patients had metastasis in the central and lateral compartments. Multifocal lesions (both lobes or single affected lobe) were observed in 8 of 14 patients (Table2).

**Table 2:** Status of lymph node metastasis of positive right upper paraesophageal lymph node metastases according to primary tumor location

		**Lateral(−)**	**Lateral(+)**
Central(−)	Upper	0	0
	Middles	0	1
	Lower	0	0
	Multifocal in both lobes	1	0
	Multifocal in affected lobe	0	0
Central(+)	Upper	1	2
	Middle	0	0
	Lower	0	2
	Multifocalin both lobes	2	4
	Multifocal in affected lobe	1	0

Univariate analysis of the clinicopathologic factors associated with metastasis to a right upper paraesophageal lymph node (Table3) indicated that right upper paraesophageal lymph node metastasis was significantly associated with larger primary tumors, multifocal tumors, extrathyroidal extension, and lymphatic invasion (*p <*0.05 each factor). Although the number of removed lymph nodes was not statistically significant, the numbers of metastatic central lymph nodes and lateral lymph nodes in the patients with right upper paraesophageal lymph node metastasis were statistically significant. In contrast, age, gender, tumor location, necrosis, vascular invasion, and perineural invasion were not associated with the occurrence of right upper paraesophageal lymph node metastasis. Although there was no statistical significance, a right upper paraesophageal lymph node metastasis originating from a PTC in the contralateral left thyroid lobe occurred in only 1 of 14 patients.

**Table 3:** Univariate analysis of the clinicopathologic factors associated with right upper paraesophageal lymphnode metastasis

		**Status of right upper paraesophageal lymph node**		***p*-Value**
		**Metastasis(−)*n*** **= 229**	**Metastasis(+)*n*** **= 14**	
Age(yrs)	Mean(95%CI^a^)	49.51(48.04-50.97)	50.07(41.11-59.03)	0.73
Gender	Male	38	5	0.07
	Female	191	9	
Tumor size	Mean(95%CI^a^)	1.0195(0.9455-1.0938)	1.6429(1.1599-2.1258)	0.006
	<1 cm	125	3	0.016
	≥1 cm	124	11	
Tumor location	Left	77	1	0.06
	Right	95	6	
	Isthmus	6	0	
	Both	51	7	
Solitary lesion	Upper	34	3	0.15
	Middle	80	1	
	Lower	41	2	
	Isthmus	6	0	
Multifocal lesion	Both lobes	13	1	
	Affected lobe	51	7	
Extrathyroidal extension	(−)	100	1	0.009
	(+)	129	13	
Lymphatic invasion	(−)	119	0	0.000
	(+)	110	14	
Multiplicity	(−)	156	4	0.002
	(+)	73	10	
Vascular invasion	(−)	219	13	0.49
	(+)	10	1	
Combined thyroiditis	(−)	185	13	0.39
	Hashimoto thyroiditis	27	0	
	Lymphocytic thyroiditis	17	1	
Retrieved central lymph nodes	Mean(95%CI^a^)	8.2664(7.5873-8.9454)	9.2143(6.0666-12.3619)	0.33
Metastatic central lymph nodes	Mean(95%CI^a^)	1.3493(1.0205-1.6782)	4.9286(2.5700-7.2872)	0.004
Retrieved lateral lymph nodes	Mean(95%CI^a^)	20.08(17.0944-23.0656)	18.6(13.2234-23.9766)	0.64
Metastatic lateral lymph nodes	Mean(95%CI^a^)	0.2882(0.1709-0.4055)	3.5(1.4029-5.5971)	0.000

^a^CI: confidence interval.

Recurrent laryngeal nerve injury was observed in 6 of 243 patients. Permanent hypoparathyroidism requiring calcium and vitamin D supplementation occurred in 24 (9.9%) of 243 patients. Postoperative whole-body radioiodine scanning and determination of rhTSH-stimulated serum thyroglobulin concentration were performed in 182 (74.9%) patients, and no visible iodine uptake was seen outside the thyroid bed. The stimulated thyroglobulin levels were low or undetectable.

## Discussion

The appropriate extent of cervical lymph node dissection in PTC remains controversial [12]. Some authors have concluded that if the tumor is smaller than 10 mm and the patient belongs to a low-risk group, the good prognosis of these patients allows for conservatives urgical treatment. They have found that extensive lymph node dissection offers no advantage in the treatment of PTC and increases morbidity [2,13,14]. Other authors have recommended total thyroidectomy with central lymph node dissection because a high rate of locoregional recurrence and distant metastasis from PTC may decrease survival, and preoperative assessment of the central compartment by US [15-18] is less sensitive than lateral compartment ultrasound.

In this study, total thyroidectomy with comprehensive central lymph node dissection, including right upper paraesophageal lymph node dissection, was performed with or without evidence of lateral lymph node metastases on preoperative US and CT studies.

In general, cervical lymph node metastases are quite common in PTC, having been found in 20% to 50% of patients. In this study, 49.4% of the patients had cervical lymph node metastases. Most series have reported a higher rate of nodal disease in the central compartment and lateral neck than in the mediastinum [7,19]; however, right upper paraesophageal lymph node metastasis posterior to the right recurrent laryngeal nerve is associated with superior mediastinal lymph node metastasis and is a risk for invasion of the mediastinal organs, including the trachea [20,21]. Because it has been associated with a high risk of postoperative hypocalcemia [22], there are currently reduced numbers of indications for elective right upper paraesophageal lymph node dissection. However, reoperative cervical lymph node dissection can be challenging and places the regional lymph nodes and parathyroids at increased risk [5,23]. There may be a higher rate of permanent recurrent laryngeal nerve injury and permanent hypoparathyroidism when cervical lymph node dissection is performed with total thyroidectomy than for total thyroidectomy alone [5].The rate of permanent hypoparathyroidism has ranged from 0% to 14.3%, and the rate of unintentional recurrent laryngeal nerve injury has ranged from 0% to 5.7% [9,16,23-25]. The results of this study were similar. Permanent recurrent laryngeal nerve injury and permanent hypoparathyroidism were found in 2.5% and 9.9% of patients, respectively. The morbidity-to-benefit ratio remains unclear for central lymph node dissection, but it can be performed safely by expert surgeons.

Zhang et al. [26] demonstrated that primary tumors arising from the upper lobe of the thyroid spread frequently to the lateral nodes while skipping the central nodes, whereas primary tumors originating from the lower lobe spread directly to the central nodes. Although the study of Zhang et al. was important for the understanding of nodal spreading, our study did not find an association between the location of the primary tumor and metastatic status of neck lymph nodes in patients with right upper paraesophageal lymph node metastasis.

In this study, 14 of 243 patients who underwent thyroidectomy with cervical lymph node dissection had right upper paraesophageal lymph node metastasis. Univariate analysis showed that larger tumor size, multifocal tumors, extrathyroidal extension, and lymphatic invasion were associated with right upper paraesophageal lymph node metastasis. Of the 14 patients, 2 were found with skip metastasis. Two of 14 patients had lymph node metastasis without central compartment metastasis, and 1 of these patients had lateral lymph node metastasis. In these two cases, the tumor size was greater than 2 cm, cancer was observed in both lobes, and extrathyroidal extension was observed. Although there was no statistical significance, a right upper paraesophageal lymph node metastasis originating from a PTC in the contralateral left thyroid lobe occurred in only 1 of 14 patients. The tumor size was 2.4 cm, multifocal cancer was observed in the affected lobe, and extrathyroidal extension was observed. According to the preoperative study and operative findings, if a patient only has cancer in the left thyroid lobe, a right upper paraesophageal lymph node dissection should be performed.

Patients with nodal metastasis from papillary carcinoma of the thyroid have an increased rate of extrathyroidal invasion, locoregional recurrence, and distant metastasis [27-29]. Extrathyroidal extension is also known to be a potent and adverse prognostic indicator [1,30]. Lymphatic and vascular invasion are markers of aggression and carry a less favorable long-term prognosis [31-33]. Thus, if thyroid cancer shows these features (multiplicity, extrathyroidal extension) during surgery, it may be necessary to inspect the right upper paraesophageal lymph nodes.

This study has demonstrated that papillary thyroid carcinoma can metastasize, although rarely, solely to the right upper paraesophageal lymph nodes posterior to the recurrent laryngeal nerves. Because of the relatively brief initial follow-up period, local recurrence, distant metastasis, and prognosis were not evaluated. The authors plan on reporting more comprehensive results and ample data, which will be collected during long-term-follow up.

## Conclusions

Right upper paraesophageal lymph node metastasis posterior to the recurrent laryngeal nerves from papillary thyroid carcinoma is fairly uncommon. In univariate analysis, metastasis to these lymph nodes was associated with tumor size, multiplicity, extrathyroidal extension, and lymphatic invasion. The results suggest that for locoregional control, it would be helpful to examine the right upper paraesophageal lymph nodes during surgery for patients with extensive cervical lymph node metastases from PTC.

## Competing interests

The authors declare that they have no competing interests.

## Authors’ contributions

WCP: participated in the conception and design of the study and carried out the surgery. YSK: participated in data analysis and interpretation of data drafted the manuscript. All authors read and approved the final manuscript.
